# Timing of Carpal Tunnel Syndrome Treatment Based on Social Deprivation

**DOI:** 10.7759/cureus.75894

**Published:** 2024-12-17

**Authors:** Akhil Dondapati, Callista Zaronias, Janet N Tran, Cody C Fowler, Thomas J Carroll, Bilal Mahmood

**Affiliations:** 1 Department of Orthopedic Surgery, University of Rochester Medical Center, Rochester, USA

**Keywords:** area deprivation index, carpal tunnel, outcomes, social deprivation, surgery

## Abstract

Introduction: This study sought to investigate the impact of the area deprivation index (ADI) on the treatment timeline from carpal tunnel syndrome (CTS) to carpal tunnel release (CTR). We hypothesize that increased social deprivation will correlate with increased time between care milestones from presentation to surgery.

Methods: This is a retrospective review of patients diagnosed with CTS who underwent CTR at a single academic institution. Variables including gender, race, ethnicity, smoking status, medical comorbidities, ADI, timing of visits and surgery, and electrodiagnostic (EDX) studies were collected. The analysis included univariate chi-square tests, ANOVA, and multivariate linear and logistic regressions.

Results: In total, 501 patients were divided by ADI national percentiles from least to most deprived tertiles. Univariate analysis demonstrated increased time from EDX to CTR comparing the least and most deprived tertiles (52 days vs. 95 days). On multivariate analysis, this correlation was no longer significant. Multivariate analysis also revealed a non-significant trend towards least deprived ADI correlating with a trial of corticosteroid injections. Injections prior to surgery correlated with an increased time from EDX to CTR and time from initial presentation to CTR. A diagnosis of severe CTS on EDX correlated with a decreased likelihood of corticosteroid injections.

Conclusions: Although previous studies have demonstrated mixed outcome results in CTS, we found that social deprivation does not correlate with delays in the treatment timeline. Factors other than delays in the treatment timeline may be contributing to the potentially worse outcomes in CTS patients with greater social deprivation.

## Introduction

Carpal tunnel syndrome (CTS) is the most common type of peripheral nerve entrapment encountered in the general population [1]. Within the United States itself, it has a reported prevalence of 50 per 1,000 people and an incidence of one to three per 1,000 people annually [2]. The annual economic burden of CTS has been estimated between $2.7 and $4.8 billion in the Medicare patient population alone [3]. CTS often initially presents with intermittent, nocturnal paresthesias in a median nerve distribution that increases in frequency and can progress to weakness and thenar atrophy over time [4]. These symptoms are often debilitating and can negatively impact patients’ functions and abilities to work [5,6]. CTS can be treated initially with conservative management, such as bracing and corticosteroid injections, but are often ultimately managed with carpal tunnel release (CTR) [7,8].

A patient’s socioeconomic status has been shown in numerous studies to play a role in access to healthcare, quality of life, patient outcomes, and perception of clinical bias [9,10]. The orthopedic population is no exception in relation to these trends. Studies have shown reduced access to care in patients with lower socioeconomic status with regard to ankle fractures, meniscus tears, flexor tendon injuries, and rotator cuff tears, as well as worse post-operative outcomes in total joint and spine surgery [11-14]. One measure of socioeconomic status and social deprivation is known as the area deprivation index (ADI), which takes into account rankings of neighborhoods based on their respective socioeconomic disadvantage in a specific region. ADI is represented as percentiles with higher ADI indicating greater social deprivation. It has been shown to capture variability in social deprivation in orthopedic patients at both the state and national levels [15]. Orthopedic surgeons need to consider a patient’s social environment as it can influence both the patient physical and mental health, in addition to their prognosis [16].

Previous studies in CTS have examined the relationship between ADI and the severity at presentation or post-operative patient-reported outcomes [17-21]. Results from these have been mixed. Bernstein et al. and Wright et al. demonstrated worse patient-reported outcomes with higher levels of deprivation while initially seeking care for CTS, while Aloi et al. showed no such difference [17,18,20]. Zhang et al. also found no correlation between ADI and post-operative patient-reported outcomes at three-month follow-up, although a higher distressed communities index (DCI) did show poorer pre-operative scores [19]. Given the lack of consensus and the widespread prevalence of CTS, more studies are warranted to further elucidate and expand upon the relationship between ADI and CTS treatment.

Despite ADI correlating with delayed access to healthcare and the likelihood of surgical intervention, there is a paucity of literature in its translation to CTS and CTR. This study aimed to investigate the impact of ADI on the treatment timeline for CTS to CTR. We hypothesize that increased social deprivation would correlate with increased time between care milestones from presentation to surgery.

## Materials and methods

The University of Rochester Office of Human Subject Protection institutional review board (IRB) approved this study (STUDY00000872). A waiver of consent was granted owing to the retrospective nature of the study. We performed a retrospective review of adult (≥18 years of age) patients who underwent carpal tunnel surgery at a single large academic institution between 2016 and 2023. Patients were identified by Current Procedural Terminology (CPT) code 64721. Excluded patients were those treated in the setting of trauma or who had an ipsilateral revision surgery. A total of 4,337 patients who met inclusion criteria were identified. Random sampling was utilized to select a study population of 201 patients [22]. The patients were identified from the electronic medical record and demographics, baseline characteristics, timing of visits and interventions, and electrodiagnostic (EDX) study results were collected by the study authors and compared. Social deprivation was evaluated with the ADI, a multi-faceted measure of socioeconomic disadvantage developed by Kind et al. that is validated at the Census block group level [21]. Patient zip codes were identified via chart review and then entered into a publicly available national ADI calculator. Patient ADI was grouped into tertiles to facilitate comparison and maximize power within each group in our patient population. We verified the specific diagnostic timeline by chart review, encompassing the time of first mention of CTS, timing of referral to hand surgery, time of hand surgery consultation, EDX, determination of surgical need, and date of surgery.

Descriptive statistics, including proportions, medians, and interquartile ranges summarized the data for the overall population and each subgroup. We conducted univariate analyses with chi-square tests for categorical variables and ANOVA for continuous variables. Post-hoc Tukey’s tests were performed for pairwise comparisons of time between care milestones between tertiles. Multivariate linear regression analyzed continuous time-based outcomes, and multivariate logistic regression was performed for analysis of categorical outcome variables [23]. The multivariate analysis controlled for multiple factors including demographics, relevant past medical history, and EDX results. ADI was a continuous variable in the multivariate analyses. Statistical significance was defined as p ≤ 0.05. We performed all analyses using Stata 18 (StataCorp, College Station, TX).

## Results

A total of 501 patients were included in the study, divided into tertiles based on their ADI. There were 42 patients in the most deprived tertile, 216 in the middle, and 243 in the least deprived. The cohort consisted of 207 (41.3%) males. There were no significant differences found in the baseline demographics of each group, including sex, ethnicity, diabetes, thyroid disease, number of patients with EDX prior to presentation, number of patients who received steroid injections, and EDX severity. However, we did find significantly more patients who were black and smokers in the least deprived ADI tertile (Table 1).

**Table 1:** Comparison of patient characteristics by thirds of national area deprivation index (ADI) Data are presented as Number (%) or Median (Interquartile Range) as appropriate. EDX - Electrodiagnostic studies

	Overall	ADI, 1-33	ADI, 34-66	ADI, 67-100	Overall, p-value	Low vs. middle, p-value	Low vs. high, p-value	Middle vs. high, p-value
Patients	501	42	216	243	-	-	-	-
Male (%)	207 (41.3)	17 (40.5)	91 (42.1)	99 (40.7)	0.949	-	-	-
Race (%)	-	-	-	-	0.013	-	-	-
Caucasian	430 (85.8)	38 (90.5)	195 (90.3)	197 (81.1)	-	-	-	-
Black	40 (8.0)	2 (4.8)	8 (3.7)	30 (12.4)	-	-	-	-
Other	31 (6.2)	2 (4.8)	13 (6.0)	16 (6.5)	-	-	-	-
Ethnicity – Hispanic (%)	22 (4.4)	0 (0)	8 (3.7)	14 (5.8)	0.196	-	-	-
Smoking (%)	-	-	-	-	<0.001	-	-	-
Active	70 (14.0)	1 (2.4)	22 (10.2)	47 (19.4)	-	-	-	-
Former	199 (39.8)	11 (26.2)	94 (43.5)	94 (38.8)	-	-	-	-
Never	231 (46.2)	30 (71.4)	100 (46.3)	101 (41.7)	-	-	-	-
Diabetes (%)	112 (22.4)	6 (14.3)	44 (20.4)	62 (25.5)	0.177	-	-	-
Thyroid disease (%)	98 (19.6)	11 (26.2)	39 (18.1)	48 (19.8)	0.475	-	-	-
EDX prior to presentation (%)	201 (40.1)	15 (35.7)	77 (35.7)	109 (44.9)	0.11	-	-	-
EDX severity (%)	-	-	-	-	0.06	-	-	-
Abnormal	14 (2.9)	2 (4.9)	3 (1.5)	9 (3.8)	-	-	-	-
Mild	77 (15.9)	3 (7.3)	40 (19.4)	34 (14.4)	-	-	-	-
Mild to moderate	13 (2.7)	0 (0)	7 (3.4)	6 (2.5)	-	-	-	-
Moderate	108 (22.3)	11 (26.8)	38 (18.5)	59 (24.9)	-	-	-	-
Moderate to severe	83 (17.2)	8 (19.5)	44 (21.4)	31 (13.1)	-	-	-	-
Severe	179 (37.0)	17 (41.5)	67 (32.5)	95 (40.1)	-	-	-	-
Steroid injections (%)	83 (16.6)	7 (16.7)	27 (12.5)	49 (20.2)	0.088	-	-	-
Time to EDX after presentation (days)	33 (20, 50)	28 (16, 49)	31.5 (20, 49.5)	33 (24, 54)	0.579	0.786	0.596	0.995
Time from prior EDX to presentation (days)	54 (28, 160)	36 (27, 78)	53 (30, 128)	60 (28, 265)	0.07	0.936	0.097	0.305
Time from presentation to surgery (days)	72 (34, 153.5)	75.5 (30, 171)	70 (34, 146)	72.5 (35, 161)	0.475	0.993	0.497	0.734
Time from EDX to surgery (days)	84.5 (38, 214)	51.5 (27.5, 152)	78 (40. 177)	95 (40, 264)	0.009	0.783	0.022	0.076

On univariate analysis, there was a significantly increased time from EDX to CTR in the most deprived tertile compared to the least deprived (95 days vs. 52 days, p=0.022). There was no difference when comparing the highest, middle, and lowest tertiles for time from presentation to EDX (33 days vs. 32 days vs. 28 days, p=0.596), days from prior EDX to presentation (60 days vs. 53 days vs. 36 days, p=0.07), or time from presentation to surgery (73 days vs. 70 days vs. 76 days, p=0.475) (Table 1).

On multivariate analysis, the correlation of least deprived ADI with a longer duration between EDX to CTR was a non-significant trend (1.65 days, p=0.066). There was also a non-significant trend towards least deprived ADI correlating with a trial of corticosteroid injections (OR 1.01, p=0.068). Corticosteroid injections prior to surgery correlated with an increased time from EDX to CTR (179.13 days, p<0.001) and time from initial presentation to CTR (230.77 days, p<0.001). Additionally, a diagnosis of severe CTS on EDX correlated with a significantly decreased likelihood of corticosteroid injections (OR 0.25, p=0.006). EDX being completed prior to presentation expedited care from presentation to surgery (101.78 days, p<0.001). A full breakdown of the multivariate analyses is seen in Table 2.

**Table 2:** Multivariate analysis of time elapsed between presentation and carpal tunnel surgery, electrodiagnostic studies and surgery, and trial of steroid injections EDX - Electrodiagnostic studies

	Time from presentation to surgery			Time from EDX to surgery			Trial of steroid injection		
	Coefficient	95% Conf. Int.	P-value	Coefficient	95% Conf. Int.	P-value	Odds ratio	95% Conf. Int.	P-value
ADI national percentile	0.227	[-0.796, 1.251]	0.663	1.645	[-0.107, 3.397]	0.066	1.013	[0.999, 1.026]	0.068
Male	1.123	[-39.458, 41.704]	0.957	-36.276	[-106.196, 33.643]	0.308	0.783	[0.459, 1.336]	0.369
White	1.793	[-87.033, 90.619]	0.968	18.453	[-137.912, 174.819]	0.817	0.884	[0.295, 2.643]	0.825
Black	58.68	[-53.559, 170.920]	0.305	66.713	[-127.726, 261.152]	0.5	1.013	[0.257, 4.002]	0.985
Hispanic ethnicity	4.231	[-100.638, 109.1]	0.937	-70.795	[-248.224, 106.633]	0.433	0.286	[0.057, 1.446]	0.13
Thyroid disease	11.149	[-38.763, 61.061]	0.661	-35.945	[-121.498, 49.610]	0.409	0.932	[0.486, 1.786]	0.832
Diabetes	12.346	[-35.009, 59.702]	0.609	-17.083	[-97.940, 63.774]	0.678	0.892	[0.470, 1.694]	0.728
Mild EDX	80.01	[-20.872, 180.892]	0.12	126.825	[-56.752, 310.402]	0.175	0.914	[0.324, 2.582]	0.865
Moderate EDX	31.265	[-65.095, 127.626]	0.524	73.487	[-102.190, 249.165]	0.411	0.894	[0.332, 2.408]	0.824
Severe EDX	37.765	[-54.507, 130.036]	0.422	27.925	[-142.004, 197.854]	0.747	0.252	[0.094, 0.678]	0.006
Steroid injection	230.66	[176.366, 284.955]	<0.001	179.130	[86.239, 272.021]	<0.001	-	-	-
EDX prior to presentation	-101.779	[-141.948, -61.61]	<0.001	-	-	-	0.608	[0.360, 1.029]	0.064

## Discussion

Over the years, there has been an increase in awareness of the influence of socioeconomic status and ADI on healthcare disparities and patient outcomes. The role of ADI in the hand surgery population, and more specifically CTS patients, has been controversial in the literature and the timeline of treatment modalities has not been explored. In our patient cohort, there was a significantly increased time from EDX to CTR in the most deprived ADI tertile, but this relationship was non-significant upon multivariate analysis. Completion of EDX prior to presentation to a hand surgeon showed faster time to CTR. Additionally, corticosteroid injections prior to surgery correlated with more days from EDX and from initial presentation to CTR. Lastly, patients who had severe CTS had a significantly decreased likelihood of having a corticosteroid injection.

One paper investigating the effect of social deprivation on the incidence of CTS surgery demonstrated that in England, the Index of Multiple Deprivation, similar to ADI, was correlated to the incidence of both carpal and cubital tunnel release [24]. The rate of CTR was significantly lower in the three least deprived quintiles (14.8%, 13.7%, 16.4%) compared to the most deprived quintile (21.1%) [24]. Interestingly, our study showed no delays in care correlating to social deprivation, although we did show a non-significant trend of least-deprived ADIs correlating with receiving corticosteroid injections. A higher rate of surgical intervention has been suggested to be due to more deprived groups allowing for their symptoms to become more severe before presentation, or perhaps they may have more medicolegal claims [25]. However, we did not see a difference in EDX severity between our groups.

Although results are mixed, CTS outcome measures may be influenced by social deprivation. Bernstein et al. found that when patients are initially seeking care for CTS, less deprived ADIs were associated with worse Patient-Reported Outcomes Measurement Information System (PROMIS) upper extremity (UE) and pain interference (PI) scores, but not PROMIS physical function (PF) or depression at both the state and national levels [17]. Wright showed similar worse PROMIS scores in more deprived areas [20]. By contrast, Aloi et al. showed no difference between the national ADI quartile and CTS-6 or Boston CTS questionnaire scores [18]. Lastly, Zhang et al. showed conflicting results within the study itself, with no difference in pre- or post-operative outcomes based on ADI, but a significant difference in pre-operative outcomes with more deprived DCI [19]. ADI is determined from a patient’s address and theoretically has a greater capacity to be more granular than DCI [26]. Our study suggests that ADI does not contribute to delays in care for CTS. If there are in fact decreased patient-reported outcomes in more disadvantaged populations, the patients’ perceptions do not appear to be influenced by their time to EDX or surgical care. Factors other than delays in treatment may be contributing to their possibly worse outcomes.

The location of this study’s academic institution is New York State, which has access to expanded Medicaid and a large number of surgeons, both of which increase healthcare access [27,28]. Although we looked at national ADIs, increased healthcare access in our region may explain why the patients with less deprived ADIs had similar time to care as those with more deprived ADIs. Further, multicenter studies are warranted to help mitigate this potential bias within state lines.

We found that corticosteroid injections before surgery were associated with a longer time from both EDX and initial presentation to CTR and that patients with severe CTS on EDX were less likely to receive an injection. These results are consistent with observations from prior studies. Fakunle et al. demonstrated that there is an increased risk of surgical site infections, wound complications, and one-year reoperation rates in patients who received a steroid injection pre-operative within 30 days of a CTR [29]. For this reason, hand surgeons are likely to delay surgery after an injection. EDX severity is an important prognostic factor for long-term steroid injection efficacy, with shorter time to treatment failure with severe EDXs [30]. As a result, severe patients may not be recommended to receive an injection as frequently.

This study is not without limitations. Due to the retrospective nature of the study, there is always concern for selection bias. There is a possibility for confounding factors that affect a patient’s decision for time to operative CTR versus conservative management that were not controlled for. Additionally, different surgeons within our system have different protocols and decision-making tactics with regard to when a patient is indicated for surgical intervention. We did see significantly more patients who were black and active smokers in the least deprived ADI tertile, but all other baseline demographics observed were similar. Since the study required a review of electronic medical charts, we only have access to the patient’s address recorded in the system. If the patient’s address is inaccurate or changed, we would not be able to account for this while determining ADI. Our study only used patients from a single medical institution in New York State, which may not make the results generalizable to all populations. Although ADI has been validated in prior studies, it is ultimately an estimate and may not be fully representative of an individual patient’s true socioeconomic status.

## Conclusions

Recent emphasis has been placed on the importance of social deprivation and ADI to ensure high-quality and equal access to healthcare in all populations. Although previous studies have demonstrated mixed outcome results in CTS, we found that social deprivation does not correlate with delays in the treatment timeline. Specifically, there was no significant increase in time from EDX to CTR nor time from presentation to CTR based on ADI tertile. Factors other than delays in the treatment timeline may be contributing to the potentially worse outcomes in CTS patient populations with greater social deprivation.
